# Fibromyalgia patients with elevated levels of anti–satellite glia cell immunoglobulin G antibodies present with more severe symptoms

**DOI:** 10.1097/j.pain.0000000000002881

**Published:** 2023-03-22

**Authors:** Emerson Krock, Carlos E. Morado-Urbina, Joana Menezes, Matthew A. Hunt, Angelica Sandström, Diana Kadetoff, Jeanette Tour, Vivek Verma, Kim Kultima, Lisbet Haglund, Carolina B. Meloto, Luda Diatchenko, Eva Kosek, Camilla I. Svensson

**Affiliations:** a Department of Physiology and Pharmacology, Centre for Molecular Medicine, Karolinska Institutet, Stockholm, Sweden; b Department of Clinical Neuroscience, Karolinska Institutet, Stockholm, Sweden. Sandström is now with the Athinoula A. Martinos Center for Biomedical Imaging, Massachusetts General Hospital, Harvard Medical School and Department of Radiology, Massachusetts General Hospital, Boston, MA, United States. Tour is now with the Oncology Surgery Department, Blekinge Hospital, Karlskrona, Sweden; c Faculty of Dental Medicine and Oral Health Sciences, Department of Anesthesia, Faculty of Medicine and Health Sciences, Alan Edwards Centre for Research on Pain, McGill University, Montreal, QC, Canada; d Department of Medical Sciences, Uppsala University, Uppsala, Sweden; e Division of Orthopaedic Surgery, Department of Surgery, McGill University, Montreal, QC, Canada; f Department of Surgical Sciences, Uppsala University, Uppsala, Sweden

**Keywords:** Autoimmunity, Autoantibodies, Dorsal root ganglia, Fibromyalgia, Satellite glia cells, Neuron

## Abstract

Transferring fibromyalgia patient immunoglobulin G (IgG) to mice induces pain-like behaviour, and fibromyalgia IgG binds mouse and human satellite glia cells (SGCs). These findings suggest that autoantibodies could be part of fibromyalgia pathology. However, it is unknown how frequently fibromyalgia patients have anti-SGC antibodies and how anti-SGC antibodies associate with disease severity. Here, we quantified serum or plasma anti-SGC IgG levels in 2 fibromyalgia cohorts from Sweden and Canada using an indirect immunofluorescence murine cell culture assay. Fibromyalgia serum IgG binding to human SGCs in human dorsal root ganglia tissue sections was also assessed by immunofluorescence. In the cell culture assay, anti-SGC IgG levels were increased in both fibromyalgia cohorts compared with control group. Elevated anti-SGC IgG was associated with higher levels of self-reported pain in both cohorts, and higher fibromyalgia impact questionnaire scores and increased pressure sensitivity in the Swedish cohort. Anti-SGC IgG levels were not associated with fibromyalgia duration. Swedish fibromyalgia (FM) patients were clustered into FM-severe and FM-mild groups, and the FM-severe group had elevated anti-SGC IgG compared with the FM-mild group and control group. Anti-SGC IgG levels detected in culture positively correlated with increased binding to human SGCs. Moreover, the FM-severe group had elevated IgG binding to human SGCs compared with the FM-mild and control groups. These results demonstrate that a subset of fibromyalgia patients have elevated levels of anti-SGC antibodies, and the antibodies are associated with more severe fibromyalgia symptoms. Screening fibromyalgia patients for anti-SGC antibodies could provide a path to personalized treatment options that target autoantibodies and autoantibody production.

## Abstract 2

Fibromyalgia patients with worse pain have elevated levels of anti–satellite glia cell antibodies, suggesting that a subset of fibromyalgia is driven by autoantibodies.

## 1. Introduction

Fibromyalgia (FM) is a prototypical condition of nociplastic pain, and despite a prevalence of 2% to 4%, the pathogenesis is unclear. Fibromyalgia is characterized by chronic widespread pain, hypersensitivity to touch and temperature, fatigue, cognitive difficulties, elevated nociceptive neuron sensitivity and activity, and reduced intraepidermal nerve fiber density.^6,10,12,23,27,41^ Because of the unclear pathogenesis of FM, treatment options have unsatisfactory efficacy, and diagnostic tests do not exist. In the case of FM, as with other nociplastic pain conditions, ie, conditions characterized by altered nociception that is not fully explained by nociceptive or neuropathic pain mechanisms,^14,26^ a key question is what causes the altered nociception.

To identify potential drivers of nociplastic pain in FM, we used a passive transfer mouse model to investigate a role for pathogenic, pain-driving immunoglobulin G (IgG) antibodies.^17^ IgG can induce pain independent of overt inflammation or nerve damage,^5,25,51^ and a role for antibodies has been identified in other nociplastic pain conditions like complex regional pain syndrome.^11,18,30^ We found that transferring FM IgG into mice induces pain-like behaviour, nociceptor sensitization and hyperactivity, and decreased intraepidermal nerve fiber density,^17^ which are characteristics present in some FM patients.^12,41^ Finally, we found that FM IgG accumulated in the dorsal root ganglia (DRG). Fibromyalgia IgG bound satellite glia cells (SGCs) in vivo*,* in vitro and in human DRG tissue sections.^17^ Satellite glia cells envelop sensory neuron soma and are linked to nociceptor activity and pain-like behavior in models of rheumatoid arthritis,^44^ neuropathic pain^9,20,50^ and inflammatory pain.^7,36^ Taken together, these findings suggest that SGC-binding autoantibodies underlie some pathological characteristics of FM.

We previously used single-patient IgG preparations from 8 individuals or pooled IgG preparations from several individuals. Thus, the frequency of FM patients with autoantibodies, and their association with disease severity, remains unclear. To further establish the clinical relevance of our findings, the aim of this study was to determine the frequency of SGC autoantibodies in FM patients and the relation between FM autoantibodies and FM symptom severity. As we have found that pooled FM IgG binds cultured SGCs and human DRG sections,^17^ we used these approaches to evaluate the presence of anti-SGC IgG levels in individual patient and control samples. We found that individuals with FM have elevated levels of anti-SGC IgG in 2 regionally distinct cohorts. Moreover, elevated levels of anti-SGC IgG are associated with worse disease severity.

## 2. Materials and Methods

### 2.1. Study participants

All study participants were recruited before the SARS-CoV-2/COVID-19 pandemic.

#### 2.1.1. Karolinska Institutet fibromyalgia and control subjects

Age-matched (20-60 years) and sex-matched serum samples were collected from FM patients or healthy controls (HC) between 2015 and 2017 (ethical permit number: 2014/1604-31/1), and written consent was received from all individuals. Fibromyalgia subjects and HC were recruited through advertisements in the daily press and were prescreened by a telephone interview by a research nurse to ensure that they fulfilled the ACR 2011 criteria and that no exclusion criteria were present. A physical examination was performed by a specialist in rehabilitation medicine and pain relief to ensure that they fulfilled the ACR 1990 FM criteria, and the ACR 2011 criteria were reassessed at the same occasion, as well as the inclusion and exclusion criteria. Exclusion criteria were other rheumatic or autoimmune diseases, primary causes of pain other than FM, comorbid severe somatic disorders (neurological, cardiovascular, etc), severe psychiatric disorders requiring treatment, previous heart or brain surgery, substance abuse, medication with anticonvulsants or antidepressants, self-reported claustrophobia, inability to refrain from hypnotics, nonsteroidal anti-inflammatory drugs, or analgesics (acetaminophen, tramadol, codeine, buprenorphine plaster, and strong opioids) before study participation, specifically 48 hours before the visit, hypertension (>160/90 mm Hg), obesity (body mass index >35), smoking (>5 cigarettes/day), magnetic implants, pregnancy, and the inability to understand and speak Swedish. Out of a larger sample of subjects (collected for other analyses), a representative subsample of 30 FM patient and 29 HC serum samples were tested for anti-SGC and anti-neuron antibodies.

#### 2.1.2. McGill University fibromyalgia and control subjects

Fibromyalgia and HC subjects' plasma was collected at the McGill University rheumatology clinic, the Alan Edwards Pain Management Unit, and the Research Institute of the McGill University Health Centre, and written consent was received from all participants (Institutional Review Board Study Number A05-M50-14B). Participants with diagnosed FM were recruited at the rheumatology clinic or the Alan Edwards Pain Management Unit of the Montreal General Hospital or through advertising with the Association de Fibromyalgie du Quebec. Additional FM subjects and HC were recruited through word of mouth, advertising in local newspapers, mass emails, and the MUHC and the McGill University bulletin boards. Inclusion criteria for all FM subjects were the ability to write and speak English or French, aged 40 years or older, and a clinical diagnosis of FM by a rheumatologist based on the ACR 2010 criteria. Exclusion criteria were uncontrolled medical or psychiatric conditions, clinical study participation that may interfere with this study, and prior or current drug and/or alcohol abuse; HCs were additionally excluded if they have been diagnosed with a chronic pain condition or have a history of depression. Thirty-six FM and 34 HC representative samples of a larger cohort (collected for other analyses) were tested for anti-SGC and anti-neuron antibodies.

#### 2.1.3. Osteoarthritis and control subjects

Knee osteoarthritis patients were recruited from the waiting list for total knee replacement at the Ortho Center, Upplands Väsby, Sweden, and control subjects were recruited through local newspaper advertisements (n = 10/group). Osteoarthritis inclusion criteria were radiographically diagnosed knee osteoarthritis and knee pain as the dominant pain symptom. Patients were excluded if they had chronic pain due to other causes or previous knee surgery. Control subjects had the same exclusion criteria, and their average weekly pain rating was <20-mm on a visual analogue scale (VAS). Serum was collected, and written consent was received from all patients (local ethical committee approval 2011/2036-31-1).

### 2.2. Study participant phenotyping

#### 2.2.1. Questionnaires

Minimum, maximum, and average global pain intensities over the past week and current pain intensities were assessed using a 100-mm VAS. The Fibromyalgia Impact Questionnaire (FIQ), assessing FM-related symptoms and disability, was completed by FM subjects.^21^ Depressive characteristics were assessed using the Beck Depression Inventory.^4^ Pain intensity in the McGill cohort was assessed with the global assessment of pain.

#### 2.2.2. Pressure pain thresholds

Pressure pain thresholds (PPTs) were assessed using a hand-held algometer (Somedic Sales AB) as previously described.^28^ A probe (1 cm^2^) was applied with steadily increasing pressure of approximately 50 kPa/second. Subjects pressed a button to indicate pain sensation, and the value was recorded. Pressure pain thresholds were assessed bilaterally at 4 anatomical sites (supraspinatus muscle, lateral epicondyle, gluteus muscle, and knee medial fat pad), and the average of all 8 assessments was used for subsequent analyses.

#### 2.2.3. Conditioned pain modulation score

Conditioned pain modulation (CPM) was evaluated with PPTs as the test stimulus and ischemic pain as the conditioning stimulus (Tourniquet test), as previously described.^13^ A baseline PPT assessment was performed at the quadriceps femoris muscle (right thigh) with the pressure algometer described above. A blood pressure cuff at the participants' upper left arm was inflated to 200 mm Hg, and participants lifted a 1 kg weight by extending their wrist until their perceived pain intensity exceed 50 mm on a 0- to 100-mm VAS scale. With the cuff inflated, the experimenter assessed the PPTs at the participants' right thigh repetitively, with at least 10 seconds between each assessment, for 4 minutes or until the participants decided to end the procedure (end PPT value). A CPM score was calculated for each participant: (end PPT value − PPT baseline)/PPT baseline. Positive scores represent inhibition, and negative scores represent pain facilitation.

### 2.3. Animals

Female BALB/cAnNRj mice (Janvier, France) aged 12 to 26 weeks were used to establish cell cultures. Mice were housed in IVC GM-500 cages (Techniplast) in a dedicated temperature control animal room with a 12-hour light/dark cycle with ad libitum access to food and water. Ethical approval was received from the Stockholm North Animal Ethics Board (4945-2018).

### 2.4. Cell culture and immunocytochemistry

Dorsal root ganglia–derived, SGC-enriched, or neuron-enriched cultures were established as previously described.^5,17,32^ Briefly, mice were deeply anaesthetized with isoflurane and euthanized by decapitation. Cervical through sacral DRGs were collected, nerve roots were removed, and DRGs were placed in cold Hank balanced salt solution. Dorsal root ganglia were then enzymatically dissociated with papain for 30 minutes and then with a collagenase II/dispase mixture for 30 minutes. Cells were then suspended in complete culture media consisting of F-12 media supplemented with 10% fetal bovine serum (Gibco) and 1x penicillin–streptomycin (Gibco), triturated, and seeded onto Nunc Lab-Tek glass chamber slides. To enrich the SGCs, the nonadherent cells, including neurons, were removed. The nonadherent cells were seeded onto Nunc Lab-Tek II CC2–treated chamber slides to establish SGC-depleted, neuron-enriched cultures. Cells were then allowed to recover for approximately 16 hours. Serum or plasma samples were diluted 1:100 in 37°C complete culture media, passed through a 0.22 μM syringe filter and incubated with the cells for 3 hours. Cells were then fixed with 4% formaldehyde and antibodies against glutamine synthase (GS) or protein gene product 9.5 (PGP 9.5) were used to identify SGCs and neurons, respectively, and an anti-human IgG antibody was used to detect human IgG. Glutamine synthase is an established marker of SGCs within intact DRGs and isolated cells^19,24,35,38,49^; across 149 serum or plasma samples tested in SGC-enriched cultures, an average of 73 cells were imaged for each sample, of which 85 ± 12% were GS positive. Protein gene product 9.5 is a panneuronal marker in tissue and DRG cell cultures.^17,29,46^ All antibody details are in Table 1. Z-stack images were collected using a Zeiss LSM800 confocal microscope operated by LSM ZEN2012 software (Zeiss).

**Table 1:** Antibody details.

Target	Fluorophore	Host	Manufacturer	Catalogue #	Dilution
GS	N/A	Rabbit	Abcam	ab73593	1:500
GFAP	N/A	Rabbit	Dako	Z0334	1:1000
NF200	N/A	Chicken	Neuromics	CH22104	1:1000
PGP 9.5	N/A	Rabbit	Cedarlane	CL7756AP	1:2000
Rabbit IgG	AF488	Goat	ThermoFisher	A11008	1:300
Rabbit IgG	Cy2	Donkey	Jackson Immunoresearch	711-225-152	1:200
Human IgG	AF594	Goat	ThermoFisher	A11014	1:300
Human IgG	Cy3	Donkey	Jackson Immunoresearch	709-165-149	1:600
Chicken IgG	Cy5	Donkey	Jackson Immunoresearch	711-175-152	1:500
Human IgG (H + L) Fab fragment	N/A	Goat	Jackson Immunoresearch	109-007-003	100 µg/mL

AF, Alexa fluor; GFAP, glial fibrillary acidic protein; GS, glutamate synthase; PGP, protein gene product.

### 2.5. Human dorsal root ganglia immunofluorescence

Fibromyalgia IgG reactivity against human DRG tissue was tested as previously described.^17^ Human DRGs were collected from 6 organ donors without a history of chronic pain following consent from the next of kin (Table 2, McGill University Health Centre REB 2019-4896). Tissue was fixed on the slides with 4% PFA, blocked with phosphate buffered saline (PBS) containing 3% normal donkey serum and 0.3% Triton X-100, and then incubated with 100 μg/mL unconjugated antihuman IgG Fab fragments (H + L, Jackson Immunoresearch, West Grove, PA). Slides were then incubated with FM or HC serum diluted 1:500 in PBS with 1% normal donkey serum and 0.1% Triton X-100. Slides were incubated with antihuman IgG antibodies, then incubated with antibodies against glial fibrillary acidic protein (GFAP) and neurofilament 200 (NF200). Glial fibrillary acidic protein is expressed by rodent and primate satellite glia cells,^34,42,49^ and NF200 is expressed in 97% of human DRG neuronal soma.^39^ The tissue was then incubated with appropriate secondaries, counterstained with 4',6-diamidino-2-phenylindole (DAPI), and cover-slipped with Prolong Gold mounting media. In one series of experiments, 28 FM and 14 HC serum samples were each tested for binding on 5 separate tissue sections from a single DRG. In a separate series of experiments, FM and HC serum IgG binding to human SGCs was tested on tissue sections from 6 DRGs (5 tissue sections per donor).

**Table 2:** Human dorsal root ganglia details.

Donor	Age	Sex	Cause of death
1	69	M	Brain hemorrhage
2	53	F	Head trauma
3	27	F	Suicide
4	38	F	Anoxia
5	52	F	Brain hemorrhage
6	47	M	Brain hemorrhage

F, female; M, male.

### 2.6. Image analysis

Individual cells were identified using Cellpose, a deep-learning neural network.^43^ Cellpose was run with Python v3.7.9, and the regions of interest of each cell were imported into FIJI using a custom Python script. For analysis, a minimum pixel intensity threshold was set at 8 × SD of the mean pixel intensity of all the images collected from a single experiment. The pixel area and integrated density of IgG and GS or PGP 9.5 above the threshold was then collected for each cell. To be considered IgG, GS, or PGP 9.5 positive, a cell had to have a minimum pixel area above the 8 × SD pixel intensity threshold. The percentage of GS-positive or PGP 9.5–positive cells that were also human IgG positive was then determined. In addition, the average integrated density across all PGP 9.5–positive or GS-positive cells from a sample was determined. Human DRG images were analyzed using the artificial intelligence–based DRGquant pipeline, as described previously.^22^ Satellite glia cells were identified as perineuronal GFAP positive cells; neuronal soma were identified as NF200-positive cells with a large diameter relative to other DRG cells and a rough circular morphology; axons were identified as NF200-positive objects that were not soma-based size and morphology; nonneuron, non-SGC cells (ex. macrophages, fibroblasts, endothelial cells) and extracellular matrix were classified as other. The pixel intensity, normalized to the background threshold, was quantified for SGCs, neuronal soma, axons, and other. The average normalized pixel intensity across 5 separate slides was calculated for each serum sample. Experimenters and DRGquant were blinded to the serum type that tissue was incubated with throughout imaging and analysis. After the analyses were complete, the brightness and contrast of images were adjusted equally between the conditions (eg, FM and HC serum) for presentation purposes.

### 2.7. Enzyme-linked immunosorbent assays

Total IgG titres were quantified using enzyme-linked immunosorbent assays (ELISA, BMS2091, Thermofisher Scientific, Waltham, MA). Enzyme-linked immunosorbent assays were performed according to manufacturers' instructions and read with a Spectramax iD3 plate reader (Molecular Devices, San Jose, CA) and analyzed with Softmax Pro software (Molecular Devices). Plate effects were corrected using a linear mixed-effects model in RStudio v1.4.1106.

### 2.8. Statistics

All data are presented as mean ± 95% confidence intervals (CI). Differences in the percentage of cells bound by IgG were analyzed by Mann–Whitney test or a Kruskal–Wallis test and Dunn post hoc test. IgG integrated density, IgG titres, and phenotypic data were analyzed by 2-tailed *t*-tests or 1-way analysis of variance and Tukey post hoc test. Spearman rank correlations were used to investigate relationships between IgG binding and phenotypic data. Differences in IgG binding to human DRG tissue across 6 donors was analyzed with a paired *t* test, where the DRGs from the same donor were matched. Karolinska FM serum samples were clustered into FM-mild and FM-severe groups using a k-means cluster analysis in RStudio v1.4.1106. A *P* value of <0.05 was considered significant. All data were analyzed in GraphPad Prism v9 except for correlations that were analyzed in RStudio v1.4.1106.

### 2.9. Data availability

Data are available from the corresponding author upon reasonable request.

## 3. Results

### 3.1. Fibromyalgia serum has increased levels of anti–satellite glia cell antibodies

**Figure 1.**
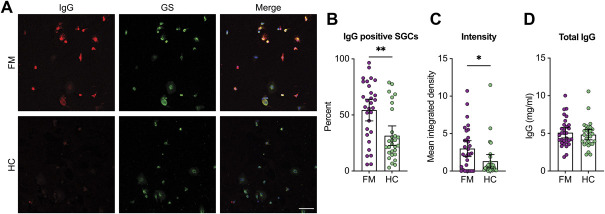
Fibromyalgia serum contains elevated levels of IgG that are reactive with satellite glia cells. Satellite glia cell–enriched cell cultures were incubated with serum from fibromyalgia (FM) or healthy control (HC) individuals and then stained for glutamine synthase (GS), a marker of SGCs. (A) Representative images of cultures incubated with FM or healthy control serum where human IgG is red and GS is green. (B) The percentage of SGCs that were bound by human IgG was determined for each serum sample tested. (C) The integrated density of IgG, as a measure of pixel intensity, was determined for each SGC that was imaged, and then, the mean integrated density was calculated for each serum sample. (D) Total IgG serum titres did not differ between the groups. Scale bar is 50 μm. N = 30 (FM) or 29 (HC); the difference between the percentage of IgG-positive cells was determined with a Mann–Whitney test, and the difference between integrated density was determined with a 2-tailed *t* test. Experimenters were blind to serum type during imaging and analysis. *Indicates *P* < 0.05, **indicates *P* < 0.01. SGC, satellite glia cell.

To evaluate levels of satellite glia cell (anti-SGC antibodies) or neuron binding IgG in FM serum, we established SGC-enriched and neuron-enriched cell cultures from mouse DRGs. Glutamine synthase, an established SGC marker,^19,24,35,38,49^ was used to identify SGCs. Across 149 serum or plasma samples tested in SGC-enriched cultures, an average of 73 cells were imaged, of which 79.9 ± 14% were GS positive. Live cell cultures were incubated with serum from single FM patient or healthy control (HC) subject so that IgG could only bind cell-surface antigens. Experimenters were blinded to serum type during imaging and analysis. Anti-SGC IgG levels were quantified by the percentage of SGCs bound by human IgG, and the binding intensity of IgG to SGCs was assessed by integrated density. IgG in FM serum bound a higher percentage of SGCs and generated a greater immunofluorescence signal intensity compared with HC serum IgG (Figs. 1A–C). These results indicate that, on average, FM serum contained higher levels of anti-SGC antibodies. Importantly, total IgG levels did not differ between the groups (Fig. 1D). When examining individual data points (Fig. 1B), we observed a large variation between individual serum samples suggesting that only a subset of FM patients have autoreactive IgG antibodies.

### 3.2. Fibromyalgia plasma has increased levels of anti–satellite glia cell antibodies in a validation cohort

**Figure 2.**
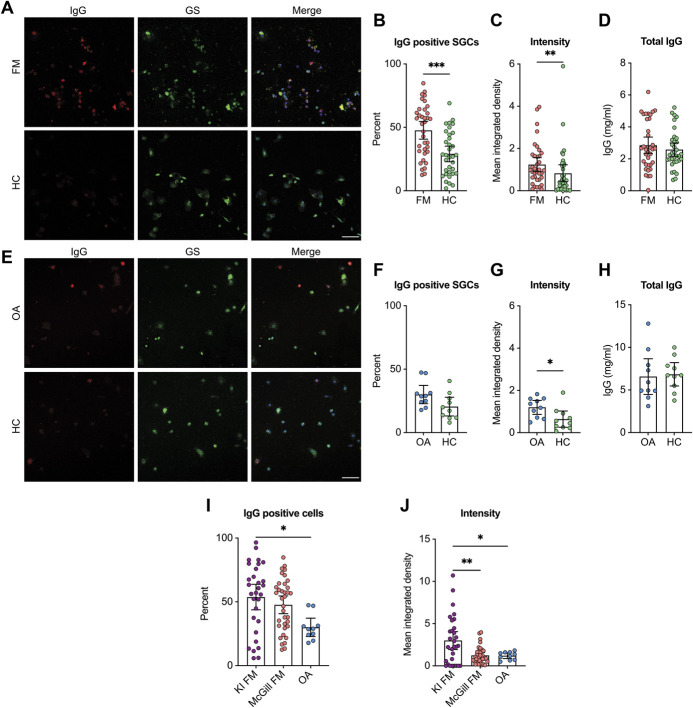
Fibromyalgia plasma contains elevated levels of SGC-reactive IgG in a validation cohort, but osteoarthritis serum does not. SGC-enriched cultures were incubated with plasma collected from a regionally distinct FM cohort. (A) Representative images of cultures where human IgG is red and glutamine synthase (GS) is green. (B) The percentage of human IgG + SGCs and (C) the integrated density (intensity) of IgG binding to SGCs were determined. (D) Total IgG plasma titres did not differ between the groups. (E) SGC-enriched cultures were incubated with serum from osteoarthritis (OA) and HC individuals. (F) The percentage of IgG + SGCs and (G) binding intensity were determined. (H) Total IgG serum titres did not differ between the groups. (I) The percentage of IgG + SGCs and (J) the binding intensity were compared between the FM and OA cohorts. Scale bar is 50 μm. N = 36 (McGill FM), 34 (McGill HC), 10 (OA), or 30 (Karolinska FM). Percentages were assed using a Mann–Whitney test (B and F) or a Kruskal–Wallis test followed by Dunn post hoc test (H). Intensities were analyzed by 2-tailed *t*-tests (C and G) or a 1-way ANOVA followed by Tukey post hoc test. Experimenters were blind to serum or plasma type during imaging and analysis. *indicates *P* < 0.05, **indicates *P* < 0.01, ***indicates *P* < 0.001. ANOVA, analysis of variance; FM, fibromyalgia; HC, healthy control; SGC, satellite glia cell.

To validate our findings, we repeated these experiments with a regionally distinct cohort of FM and HC plasma samples from the McGill University. We found that FM plasma samples had elevated levels of IgG that bound SGCs, both in terms of IgG + SGCs and the binding intensity (Figs. 2A–C). The total IgG levels did not differ in plasma between the groups (Fig. 2D). These results indicate that increased levels of anti-SGC antibodies are not specific to a single FM cohort and are present in regionally distinct cohorts.

### 3.3. Anti–satellite glia cell antibodies are not ubiquitous across chronic musculoskeletal pain

To determine whether anti-SGC IgG is widespread across chronic musculoskeletal conditions, we tested osteoarthritis patient and matched control serum. There was no difference between the percentage of SGCs bound by IgG in osteoarthritis serum compared with control serum and only a slight elevation of binding intensity (Figs. 2E–G). The total IgG level also did not differ between osteoarthritis (OA) and HC serum (Fig. 2H).

The Karolinska FM samples bound an elevated level of SGCs compared with the osteoarthritis samples (Fig. 2I), and the binding intensity of the Karolinska samples was greater than the McGill FM samples and the OA samples (Fig. 2J). This further supports that the presence of SGC-reactive IgG is not widespread across chronic pain conditions. However, this comparison also raises the interesting finding that Karolinska FM samples have, on average, a stronger binding profile than the McGill FM samples.

### 3.4. Anti-neuron IgG levels are not different between fibromyalgia and control groups

**Figure 3.**
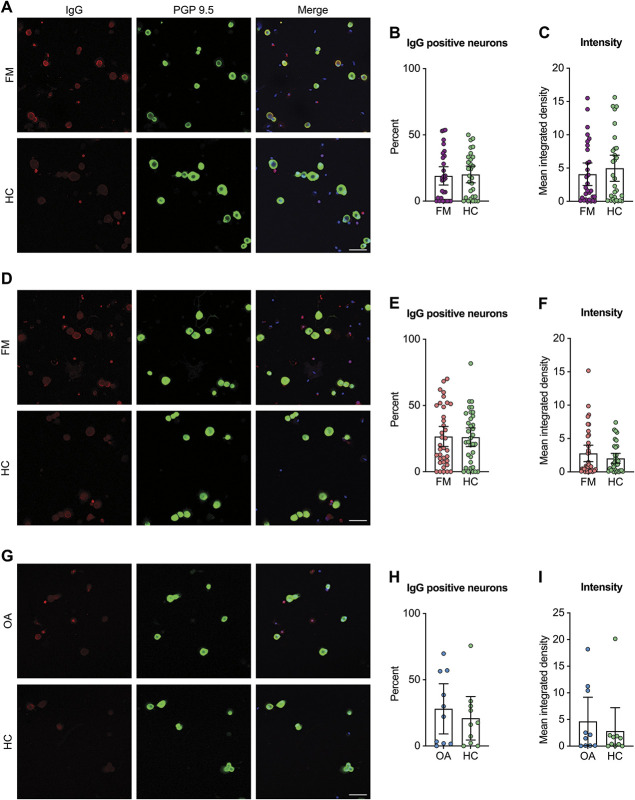
Fibromyalgia and osteoarthritis patients do not have elevated levels of sensory neuron binding IgG. Dorsal root ganglia neuron–enriched/satellite glia cell depleted cultures were incubated with serum from FM patients (A), plasma from FM patients (D), serum from osteoarthritis (OA) patients, or corresponding healthy control individuals (HC). (A, D, G) Neurons were identified by PGP 9.5 immunoreactivity (green); IgG binding was detected using an antihuman IgG antibody (red), and cultures were counterstained with Hoechst (blue). The percentage of neurons bound by IgG (B, E, H) and the intensity of the binding to neurons assessed by integrated density (C, F, I) were determined. Scale bar is 50 μm. In (A–C), n = 30 (FM) and 29 (HC); in (D–F), n = 36 (McGill FM) and 34 (McGill HC); in (G and H), n = 10 (OA and matched HC). Differences in percentages were determined using a Mann–Whitney test, and differences in intensity were determined using 2-tailed *t*-tests. Experimenters were blind to serum type during imaging and analysis. FM, fibromyalgia; PGP, protein gene product.

Next, we assessed anti-neuron IgG levels. There was no difference in the percentage of neurons bound by IgG nor the IgG binding intensity to neurons in the Karolinska FM (Figs. 3A–C), McGill FM (Figs. 3D–F), and osteoarthritis (Figs. 3G–I) samples compared with the matched control group. This suggests that the presence of neuron-binding IgG antibodies in FM serum and plasma is not widespread.

### 3.5. Anti–satellite glia cell antibody levels correlated with pain intensity

**Figure 4.**
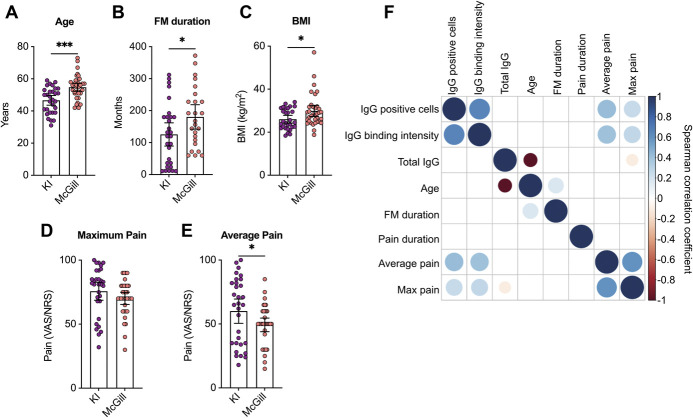
Fibromyalgia IgG binding to satellite glia cells positively correlated with pain intensity. Comparisons of (A) age, (B) FM duration, (C) body mass index (BMI) measured as time from diagnosis, (D) maximum pain, and (E) average pain. Pain intensity was assessed with the visual analogue scale (VAS) of pain at Karolinska and with the numeric rating scale (NRS) of pain at McGill. Both scales have a range of 0 to 100. (F) A correlation matrix showing correlations between the percentage of IgG-positive cells and the binding intensity with total IgG levels, age, FM duration, pain duration, and average and maximum pain intensities were examined in FM serum (Karolinska) and plasma (McGill) samples only. The size of the dots and the colour (see colour legend) indicate the Spearman rank correlation coefficient. Only statistically significant correlations (*P* < 0.05) have coloured dots, whereas correlations that were not significant are blank. Differences in age, BMI, and pain intensity were assessed by 2-way ANOVA followed by Tukey post hoc test. Differences in FM duration were analyzed by a 2-tailed *t* test. *indicates *P* < 0.05, ***indicates *P* < 0.001. N = 30 (Karolinska FM), 36 (McGill FM), 29 (Karolinska HC), 34 (McGill HC), or 66 (FM correlations). ANOVA, analysis of variance; FM, fibromyalgia; HC, healthy control.

We next investigated whether anti-SGC IgG levels in FM patients correlate with phenotypic characteristics across cohorts. Fibromyalgia patients from McGill were on average older, had a higher body mass index (BMI), and had had FM longer. The maximum pain intensity did not differ between the cohorts, but the average pain intensity was greater in Karolinska FM patients compared with McGill FM patients (Figs. 4A–E). Anti-SGC IgG levels, determined by the percentage of SGCs bound and the SGC IgG-binding intensity, did not correlate with age, BMI, total IgG, or the duration an individual has had FM or chronic pain. However, anti-SGC IgG levels positively correlated with the self-reported average pain intensity (moderate) and maximum pain intensity (moderate) (Fig. 4F). These findings suggest that anti-SGC antibody levels are associated with pain intensity.

### 3.6. Fibromyalgia Impact Questionnaire, pain thresholds, and pain intensity correlated with anti–satellite glia cell IgG levels

**Figure 5.**
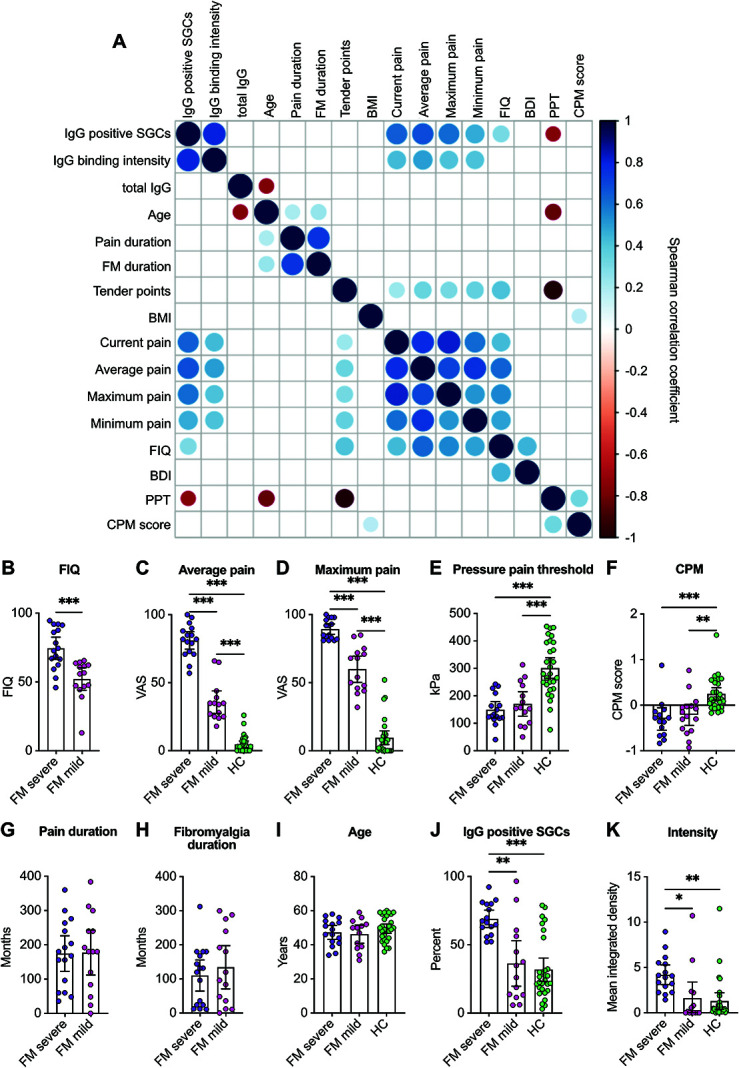
Level of satellite glia cell–reactive fibromyalgia IgG is associated with disease severity. The Karolinska cohort has expanded phenotypic data; therefore, additional correlations between IgG-positive cells and IgG binding intensity with various characteristics were examined in the FM samples. (A) Correlation matrix showing the relation between anti-SGC levels and additional phenotypic data. The size and colour (see colour legend) of the dots indicate the Spearman rank correlation coefficient, and only statistically significant correlations (*P* < 0.05) have coloured dots, whereas nonsignificant correlations are blank. The Karolinska FM cohort was divided into 2 groups based on FIQ, average pain intensity, and maximum pain intensity using a k-means cluster analysis. The cluster analysis resulted in a FM severe and a FM mild group (B-I). (J) The percentage of satellite glia cells bound by IgG and (K) the binding intensity were both greater in the FM severe group compared with the FM mild group and the healthy control group (HC). One-way ANOVAs with Tukey post hoc test (C–F, I–K) or 2-tailed *t*-tests (B, G, H) were used to examine differences between the groups. *indicates *P* < 0.05, **indicates *P* < 0.01, ***indicates *P* < 0.001. ANOVA, analysis of variance; BMI, body mass index; BDI, becks depression inventory; CPM, conditioned pain modulation; FIQ, fibromyalgia impact questionnaire; FM, fibromyalgia; PPT, pressure pain threshold; SGC, satellite glia cell.

Karolinska FM patients had additional phenotypic characterization, allowing for further analysis. When analyzing the Karolinska FM cohort alone, anti-SGC IgG levels positively correlated with the average, current, minimum, and maximum pain intensities (moderate). In addition, the percentage of cells bound by IgG positively correlated with the FIQ score (moderate) and negatively correlated with an individual's pressure pain threshold (weak). Of note, anti-SGC IgG levels did not correlate with CPM (Fig. 5A). These correlations indicate that worse disease severity is associated with elevated levels of anti-SGC antibodies.

### 3.7. Individuals with more severe fibromyalgia have elevated levels of anti–satellite glia cell IgG

The Karolinska FM cohort was split into FM-mild and FM-severe groups using a k-means cluster analysis with the average and maximum pain intensities and the FIQ score. The FM-severe group had elevated FIQ scores and pain intensities compared with the mild group and HC (Figs. 5B–D). Pressure pain thresholds, condition pain modulation scores, chronic pain duration, FM duration, and age did not differ between the FM groups (Figs. 5E–I). The FM-severe group had an increased percentage of IgG-bound SGCs, and the IgG binding intensity was greater compared with the FM-mild group (Figs. 5J and K). However, the FM-mild group did not differ from HC. These results suggest that SGC-reactive IgG levels are elevated in FM patients with more severe disease.

### 3.8. Fibromyalgia serum has elevated levels of IgG that bind human satellite glia cells

**Figure 6.**
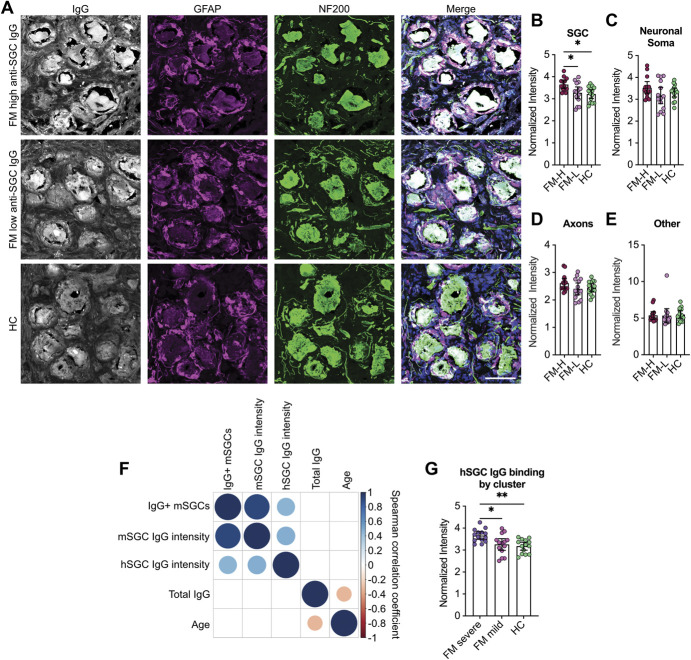
Elevated FM IgG binding to human satellite glia cells. (A) Human DRG tissue sections were incubated with serum samples that had high levels or low levels of anti-SGC antibodies detected in mouse DRG–derived SGC cultures. Human SGCs (hSGCs) were identified with an antibody against glial fibrillary acidic protein (GFAP), and neurons were identified with an antibody against NF200. The normalized pixel intensity of serum IgG binding to (B) SGCs, (C) neuronal soma, (D) axons, and (E) GFAP-negative and NF200-negative objects was analyzed. (F) The pixel intensity of IgG binding to hSGCs correlated with the level of anti-SGC antibodies determined in cell culture by the percent of IgG positive mouse SGCs (mSGCs) and the binding intensity (integrated density). The size and colour of the dots indicate the Spearman rank correlation coefficient, and only statistically significant correlations (*P* < 0.05) have coloured dots, whereas nonsignificant correlations are blank. (G) Individuals in the FM severe group had elevated IgG binding to hSGCs compared with the FM mild group and HC group. One-way ANOVAs with Tukey post hoc test was used to determine differences, and experimenters were blind to serum type during imaging and analysis. *indicates *P* < 0.05, **indicates *P* < 0.01, n = 14/group. Scale bar is 50 μm. ANOVA, analysis of variance; DRG, dorsal root ganglia; FM, fibromyalgia; HC, health control; SGC, satellite glia cell.

Next, we wanted to determine whether FM serum samples with high levels of anti-SGC IgG also bound human DRG tissue. Human DRG tissue sections were incubated with FM serum containing high or low levels of anti-SGC IgG detected in culture, or HC serum. Experimenters were blinded to serum type during imaging and analysis. Satellite glia cell–bound IgG normalized pixel intensity was greater in the serum samples with high levels of anti-SGC IgG compared with serum with low levels of anti-SGC IgG and controls, whereas there was no difference between serum with low anti-SGC IgG and HC serum (Figs. 6A and B). There were also no differences in IgG binding to neuronal soma, axons and non-SGC, nonneuronal tissues (Figs. 6C–E). The IgG binding intensity to human SGCs moderately correlated with anti-SGC IgG levels determined in SGC-enriched cultures, but there was no association with total IgG levels or age (Fig. 6F). Finally, IgG binding intensity to human SGCs in the FM-severe group was elevated compared with the FM-mild group and HC group (Fig. 6G). Together, these findings indicate that serum from patients with severe FM has elevated levels of antibodies that bind human SGCs and mouse SGCs.

### 3.9. Human satellite glia cell-expressed epitopes for fibromyalgia IgG are present across multiple individuals

**Figure 7.**
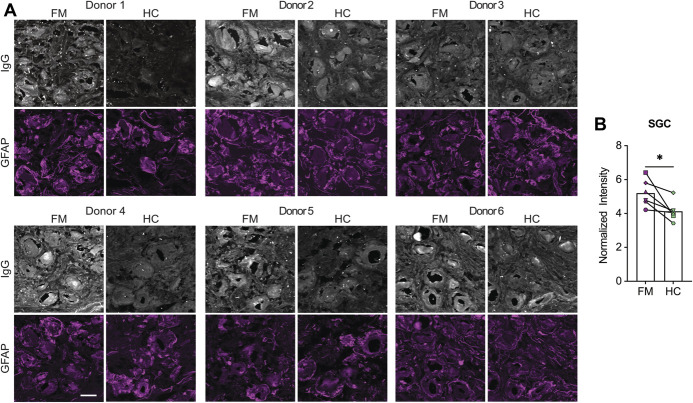
Human SGCs express epitopes for anti-SGC IgG across DRG donors. (A) DRG tissue from 6 human organ donors were incubated with an FM serum sample that had high levels of IgG against human SGCs and with an HC serum sample. Human SGCs were identified as glial fibrillary acidic protein (GFAP)–positive objects with a perineuronal location, and (B) the normalized pixel intensity of serum IgG binding to SGCs was quantified. Each symbol is the average normalized pixel intensity across 5 slides from a single DRG donor. Data were analyzed with a paired *t* test; experimenters were blind to serum type during imaging and analysis, *indicates *P* < 0.05, n = 6 DRGs/group. Scale bar is 50 μm and each symbol in (B) represents a single DRG donor. DRG, dorsal root ganglia; FM, fibromyalgia; HC, health control; SGC, satellite glia cell.

Finally, we wanted to determine whether human satellite glia cells express the epitopes for FM anti-SGC IgG across multiple individuals. We investigated whether an FM serum sample with high levels of antihuman SGC IgG had elevated binding in DRGs from 6 different organ donors (Table 2). On average, FM serum IgG had elevated binding to human SGCs compared with HC serum IgG, and 5 out of 6 donors had clearly elevated FM IgG binding to SGCs compared with HC serum (Fig. 7). These results suggest that the SGC-expressed epitopes bound by FM IgG are present across individuals.

## 4. Discussion

We previously found FM patient IgG induces FM-like characteristics in mice and that FM IgG binds mouse and human SGCs. However, the frequency of FM patients with SGC binding IgG and the correlation with clinical symptoms was not examined. Here, we found elevated anti-SGC antibodies in 2 distinct FM cohorts. Moreover, the binding of IgG to SGCs in our cell culture assay correlated with disease severity, indicating the clinical relevance of our finding. Importantly, IgG binding to human SGCs is also elevated compared with control group and correlated with anti-SGC IgG levels detected in culture. Fibromyalgia IgG binding to human SGCs was also elevated in more severe FM, further supporting a link between anti-SGC autoantibodies and disease severity. Furthermore, these results suggest that pathogenic autoantibodies either underlie FM in a subgroup of patients or are present at concentrations too low to be detected in our assays in individuals with mild disease.

When considering the heterogeneity of FM symptoms and severity, it is unsurprising that not all individuals have elevated anti-SGC IgG. Although pain is a uniting factor between the individuals, the severity of pain and the cognitive and mood symptoms can differ greatly. Numerous studies have clustered FM patients based on pain reports, and cognitive and psychological questionnaires.^3,8,16,52^ In addition, 60% of FM patients have reduced intraepidermal nerve fiber density (ie, reduced skin innervation), which is associated with more severe pain and disease.^12^ Together, these studies suggest that multiple overlapping disease mechanisms contribute to FM pathology, and targeting these mechanisms may prove beneficial therapeutically. However, questionnaire-based grouping of patients has yielded limited insight for personalized treatment, and widespread adoption of quantitative sensory testing, intraepidermal nerve fiber density measurement, and microneurography are limited by time and skill constraints. Our study suggests that FM patients could be stratified based on autoantibody detection, which could be used for more personalized treatment approaches.

Autoantibody status is used as an indicator of disease subtype and to inform treatment strategies in other autoimmune and autoantibody-mediated diseases. For example, rheumatoid arthritis is divided into seronegative and seropositive cases where seropositive individuals have rheumatoid factor, anticitrullinated protein antibodies, or both, and seronegative individuals have neither. Seropositive rheumatoid arthritis is associated with more severe joint destruction, and seropositive individuals respond better to B-cell depletion.^33^ Myositis disease subsets are also characterized by specific autoantibodies, which improve diagnosis and treatment approaches.^2,31,40^ Our results suggest that FM patients could be divided into seropositive and seronegative subsets, creating the possibility for personalized medicine strategies. To make this a reality, future studies will need to investigate whether anti-SGC antibodies are associated with the cognitive and mood symptoms of FM and FM autoantibody targets.

Fibromyalgia was long thought of as a central nervous system disease, but it is increasingly clear that the peripheral nervous system and the immune system are important contributors to FM pathology. Hyperactivity of primary nociceptors^41^ and reduced intraepidermal nerve fiber density^12^ in FM patients suggest that neuroplasticity occurs in the DRG, rather than only the brain and spinal cord. Both nociceptor hyperactivity and reduced intraepidermal nerve fiber density are induced by transferring FM IgG into mice.^17^ FM IgG also binds antigens expressed in mouse and human DRGs, further suggesting causal roles of the peripheral nervous system and immune system in FM. Peripheral immune cells, such as natural killer cells^47^ and mast cells,^45^ are also linked to FM. Our findings indicate that the interaction between the peripheral nervous system and the immune system occurs in part through autoantibodies, like those that bind SGCs. We found that anti-SGC IgG levels are elevated in individuals with more severe pain, increased pressure pain sensitivity, and higher FIQ scores but are unrelated to CPM. The lack of association with CPM suggests that anti-SGC antibodies are driving peripheral characteristics of FM. Thus, the subgroup of FM patients with anti-SGC antibodies may correspond to a group with more severe peripheral FM characteristics like nociceptor hyperactivity and reduced intraepidermal nerve fiber density.

Individuals included in both FM cohorts often had FM for many years before blood collection, and FM diagnoses are often delayed from the disease onset.^15^ The timing between onset and blood collection raises the question of whether anti-SGC antibodies are an outcome of FM or a possible contributor to disease onset and persistence. Fibromyalgia IgG injection into mice induces FM characteristics, suggesting that autoreactive IgG contributes to FM onset.^17^ Supporting this idea, we found that anti-SGC antibody levels are associated with FM severity but not with FM duration or pain duration. To determine the time frame in which FM autoantibodies develop and how autoantibody levels fluctuate with time longitudinal studies are needed. Regardless, our findings indicate that FM autoantibodies are not a result of chronic disease but instead a contributor to the disease mechanism.

In this study, we did not find a difference between FM IgG and HC IgG binding to neurons. We had previously reported elevated IgG binding to sensory neurons in vitro*,*^17^ but these experiments were done using pooled IgG preparations. When looking at the data points from single individuals in this study, many individuals have no or low levels of neuron binding IgG, but there are also individuals who have higher levels of IgG binding to neurons. It remains possible that a subset of FM patients have anti-neuron antibodies. Regardless, if autoantibodies binding SGCs are driving nociceptor hypersensitivity, then they must do so by indirectly activating SGCs or by activating neuronally expressed Fcγ receptors.^5,48^

Our assay may lack the sensitivity to detect low levels of pathogenic IgG in FM samples, and it cannot differentiate between pathogenic anti-SGC IgG and nonpathogenic, nonspecific IgG binding to SGCs. For example, 7 HC samples had relatively high (>50% of SGCs bound by IgG) IgG binding to SGCs. We reason that among pain-free individuals, with normal variation in their antibody repertoires, some may have IgG that binds SGCs. It is possible that a recent infection in these individuals could have triggered higher levels of nonspecific antibodies. Fibromyalgia patients with comorbid pain conditions were not included in the study, but in clinical practice, FM patients often present with several medical comorbidities. Therefore, our study does not represent the general FM population but instead opens the door to future studies that aim to understand mechanisms in FM with overlapping pathologies.

Similarly, serum from OA patients had elevated SGC IgG integrated density compared with HC serum, but there was no difference in the percentage of SGCs bound. Although the difference between OA and HC serum was far less pronounced than FM and HC, limited to a single parameter, and FM serum had greater levels of anti-SGC antibodies than OA serum, it is possible that some individuals with OA have low levels of anti-SGC antibodies. Our OA cohort was also relatively small, so it could be interesting to examine anti-SGC antibody levels in a larger number of patients. Although FM was an exclusion criterion in our OA subjects, up to 25% of OA patients have comorbid FM,^14^ so examining levels of anti-SGC antibodies in subjects with severe OA with and without severe FM could prove informative. Ultimately, identifying the autoantigenic targets of FM IgG will increase specificity to differentiate between FM patients with and without autoantibodies and between other pain conditions and HC subjects who have nonpathogenic anti-SGC IgG.

Our use of murine cell cultures to assess levels of anti-SGC and anti-neuron antibodies creates a potential caveat. Mouse SGCs could express antigens that are not expressed by human SGCs and vice versa. However, serum IgG binding to SGCs in human DRG tissue sections was elevated and correlated with the level of anti-SGC IgG detected in cultures. Human SGCs are transcriptionally similar to mouse SGCs according to single cell RNA-sequencing studies,^1^ as are human DRG neurons to mouse neurons,^37^ suggesting that SGCs and neurons will have similar cell surface protein repertoires. Considering that FM IgG binds both human and mouse SGCs, it is unlikely that FM IgG is binding different or at least completely unrelated targets. Once FM autoantigens are identified, antigen-based assays can be developed to address the above caveats.

We found that anti-SGC IgG levels are elevated in 2 distinct FM cohorts. The level of these antibodies correlated with a more severe clinical presentation, while being unrelated to FM duration. Moreover, the level of anti-SGC antibodies detected in culture correlated with binding to human SGCs. These results, combined with our previous findings that FM IgG induces pain-like behaviour in mice, provide a possible answer to one mechanism of nociplastic pain in FM. Taken together, the data suggest that testing FM patients for elevated levels of anti-SGC IgG may be useful to stratify patients for therapies that interfere with antibody function such as B cell depleting drugs, IVIg, or plasmapheresis.

## Conflict of interest statement

The authors have no conflict of interest to declare.

## Supplementary Material
